# AXOLOTL: an accurate method for detecting aberrant gene expression in rare diseases using coexpression constraints

**DOI:** 10.1093/bioinformatics/btag255

**Published:** 2026-05-04

**Authors:** Wenjian Xu, Yansheng Shen, Xiangfu Liu, Fei Leng, Yang Liu

**Affiliations:** Experimental Research Center, Capital Center for Children’s Health, Capital Medical University, Capital Institute of Pediatrics, Beijing 100020, China; Aegicare (Shenzhen) Technology Co., Ltd, Shenzhen, Guangdong 518000, China; Aegicare (Shenzhen) Technology Co., Ltd, Shenzhen, Guangdong 518000, China; Beijing Key Laboratory for Genetics of Birth Defects, Beijing Pediatric Research Institute, Capital Medical University, National Center for Children’s Health, Beijing 100045, China; MOE Key Laboratory of Major Diseases in Children, Capital Medical University, National Center for Children’s Health, Beijing 100045, China; Rare Disease Center, Beijing Children’s Hospital, Capital Medical University, National Center for Children’s Health, Beijing 100045, China; Aegicare (Shenzhen) Technology Co., Ltd, Shenzhen, Guangdong 518000, China

## Abstract

**Motivation:**

The assessment of aberrant transcription events in rare disease patients holds great promise for enhancing the prioritization of causative genes—a strategy already widely adopted in clinical settings to improve diagnostic accuracy. Nevertheless, the accurate identification of causal genes remains a substantial challenge.

**Results:**

We propose AXOLOTL, a novel ensemble method for identifying aberrant gene expression events in RNA expression matrices. AXOLOTL effectively accounts for gene correlation by incorporating coexpression constraints. We demonstrated the superior performance of AXOLOTL on representative RNA-seq datasets, including those from the GTEx healthy cohort, mitochondrial disease cohorts, and collagen VI-related dystrophy cohorts. Furthermore, we applied AXOLOTL to real-world cases of neurological disorders and demonstrated its ability to accurately identify aberrant gene expression and facilitate the prioritization of pathogenic variants.

**Availability and implementation:**

AXOLOTL is freely available on GitHub (https://github.com/xuwenjian85/axolotl) and Zenodo (https://doi.org/10.5281/zenodo.17940844).

## 1 Introduction

RNA-seq is an indispensable tool for identifying pathogenic DNA variants and increasing the diagnostic rate of rare disease cohorts (Cummings et al. 2017, Kremer et al. 2017, Frésard et al. 2019, Murdock et al. 2021, Yépez et al. 2022). For undiagnosed patients, causative genes can be further prioritized if both DNA and RNA supporting evidence are considered. Three types of extreme transcription events (abnormal gene expression, alternative splicing, and allele-specific expression) are detectable in RNA-seq data (Brechtmann et al. 2018, Jenkinson et al. 2020, Mertes et al. 2021, Yepez et al. 2021, Dekker et al. 2023). Given the high diversity of rare diseases, cohorts often consist of individuals with distinct disorders. However, classical differential analysis is not suitable for accurately detecting abnormal gene expression in patients with rare diseases (Love, Huber, and Anders 2014, Cummings et al. 2017, Kremer et al. 2017). Specialized computational methods have been developed to detect aberrant gene expression (Brechtmann et al. 2018, Salkovic et al. 2020, Labory et al. 2022, Salkovic et al. 2023).

Aberrant gene expression in an RNA expression matrix is an outlier detection problem. The existing methods measure the abnormalities of matrix elements in different ways. OUTRIDER uses a denoising autoencoder to control unwanted covariations in the gene expression profile, after which the autoencoder-normalized counts are statistically modeled with gene-specific negative binomial distributions (Brechtmann et al. 2018). ABEILLE uses a variational autoencoder to compute reconstruction errors, classifies normal and abnormal genes with a decision tree model in a supervised setting and quantifies anomality with an isolation forest model in an unsupervised setting (Labory et al. 2022). The OutSingle models expression matrix with log-normal distributions, controlled for confounders with an SVD-based model, and estimates *P* values for genes (Salkovic et al. 2023). OutPyR yields a novel Bayesian outlier score without controlling for confounders, but its performance is not superior to that of OUTRIDER (Salkovic et al. 2020, Salkovic and Bensmail 2021). Although recent methods are capable of controlling hidden confounders (Brechtmann et al. 2018, Salkovic et al. 2023), whether they can fully eliminate these confounders—and further, whether gene coregulation remains a potential influence—would require additional investigation.

Genome-wide coregulation can be viewed as a coexpression network of many interconnected gene modules (Langfelder and Horvath 2008, Saelens et al. 2018, Raina et al. 2023). Inspired by that, we regard the coexpression relationship as an inherent biological constraint of an individual transcriptome. A group of coexpressing genes function like a loosely connected module, exhibiting coordinated up and down fluctuations across samples. Individual genes fluctuate within the normal variational range that is determined by the coexpressing gene module across samples. For a given sample, this means the range reflects the typical expression variation of the coregulated genes in that sample context. Thus, the more a gene deviates beyond this normal variational range in a specific sample—i.e. deviates from the typical expression of other coregulated genes in that sample—the more aberrant it is in the transcription profile.

Here, we propose AXOLOTL (Aberrant gene eXpression as OutLier detectiOn using deviaTion from normaL coexpression), an unsupervised machine learning method to detect aberrant gene expression events in RNA expression matrix. AXOLOTL extends post-OUTRIDER and post-OutSingle outputs as features and formulates a multivariate, non-parametric outlier detection problem that removes residual gene–gene correlations the autoencoder could not capture. We benchmarked the AXOLOTL and existing methods using publicly available RNA-seq datasets containing aberrantly expressed genes, which included GTEx dataset of 49 tissues (GTEx Consortium 2017, Ferraro et al. 2020), a mitochondrial disease (n = 423) dataset of fibroblast samples (Yépez et al. 2022) and a Collagen VI-related dystrophy (COL6-RD, n = 36) dataset of muscle samples (Guadagnin et al. 2021). We also validated AXOLOTL’s superior performance in aberrant-expression detection on our in-house dataset of 111 blood RNA-seq samples, which included 65 controls and 46 neurological patients who had candidate splice variants.

## 2 Materials and methods

### 2.1 Overview of axolotl

AXOLOTL is an ensemble method integrating OUTRIDER, OutSingle, and the unsupervised model LOF. Its core innovation lies in extending post-OUTRIDER/OutSingle features (*osg_p* from OutSingle’s log-transformed *P*-value and *ord_p_devi* from OUTRIDER-derived log-transformed *P*-value and *z*-score) via feature engineering to formulate a multivariate non-parametric outlier detection problem.

### 2.2 Datasets

#### 2.2.1 Real datasets

**Figure 1:**
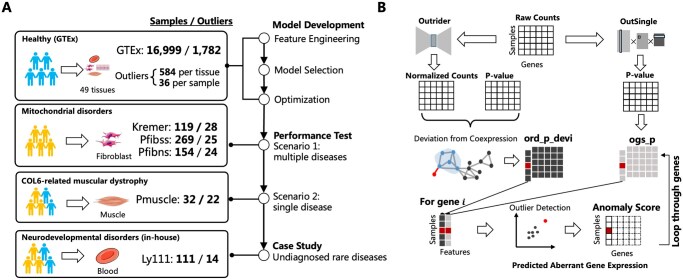
Workflow and method overview of AXOLOTL. (A) Datasets. Schematic of cohorts, tissues, RNA-seq datasets, and their roles in different stages of this study. Human shaped icons represent controls (blue) and rare disease patients (yellow). For each dataset, the samples represent the number of RNA-seq data and the outliers represent the number of sample-gene pairs with potential aberrant gene expression events. (B) AXOLOTL architecture. Input is an RNA-seq read count matrix. Rows represent samples; columns represent genes. Gene read counts are processed by baseline models, used to generate our customized features, which characterize how far each gene-sample pair deviates from its normal range. Genes correspond to columns of individual feature table (sample by gene); two features are reorganized together as gene-specific feature matrix (sample by feature). Unsupervised outlier detection model (LocalOutlierFactor) fit for one gene at a time and outputs anomaly score. Aberrant expression events are highlighted in red.

Both rare disease cohorts and healthy individual cohorts were included in this study (Fig. 1A). GTEx dataset was used for model development and optimization. The mitochondrial disease dataset (Yépez et al. 2022) and Collagen VI-related dystrophy dataset (Guadagnin et al. 2021) were used for model evaluation in the rare disease cohort. In real case studies, inherited nervous system disorders dataset were used to highlight the practical value of AXOLOTL.

##### 2.2.1.1 GTEx cohort

We processed 49 GTEx tissue RNA-seq count matrices and utilized publicly available stop-gain NMD variants from Teran et al. (2021). We identified 1782 unique predicted NMD genes, resulting in an average of 584.7 outliers per tissue and 36.3 per individual (Table 1, available as supplementary data at *Bioinformatics* online, enrich aberrant expression shown in Fig. 1A and B, available as supplementary data at *Bioinformatics* online).

##### 2.2.1.2 Rare disease cohort Kremer119

The cohort is an RNA-seq dataset of skin-derived fibroblasts from 119 patients with mitochondrial disease.

A set of 22 potential NMD expression outliers was derived from Kremer et al. (2017). Specifically, variants were annotated as “NMD” if the DNA VARIANT_EFFECT field contained “stop” or “frame-shift” terms. We then extracted entries with predicted “NMD” effect. We included six RNA-seq-validated outliers previously used in the studies (Brechtmann et al. 2018, Labory et al. 2022). In total, the Kremer119 dataset contained 28 outliers (Table 2, available as supplementary data at *Bioinformatics* online).

##### 2.2.1.3 Rare disease cohorts Pfibns154 and Pfibss269

Yépez et al. dataset, the largest publicly available rare disease RNA-seq cohort to date, is a dataset of skin-derived fibroblasts from 423 patients (including the Kremer119 dataset) suffering from mitochondrial disease or other Mendelian diseases (Yépez et al. 2022).

We compiled 49 aberrant expression events (Table 3, available as supplementary data at *Bioinformatics* online). All the outliers were reported in the aberrant expression analysis and verified by WES/WGS in original publication.

We prepared stranded and non-stranded samples separately to Pfibns154 and Pfibss269 datasets.

##### 2.2.1.4 Rare disease cohort Pmuscle36

Pmuscle36 is an RNA-seq dataset of skeletal muscle biopsies from 22 COL6-RD patients and 14 age-matched controls (Guadagnin et al. 2021).

COL6-RD is a form of congenital muscular dystrophy characterized by muscle weakness and joint defects. Pathogenic variants in collagen VI genes (COL6A1, COL6A2, and COL6A3) were found in 22 patients. Immunostaining assays indicated that COL6A proteins were aberrantly expressed or mislocalized (Guadagnin et al. 2021). We defined the COL6A genes of these patients as expression outliers (Table 4, available as supplementary data at *Bioinformatics* online). Gene read counts were produced using our in-house pipeline (Xu et al. 2022) from the RNA-seq data GSE103975.

##### 2.2.1.5 Rare disease cohort Ly111

Sixty-five controls and 46 probands suffering from inherited nervous system disorders were retrospectively enrolled. The samples were accepted and evaluated by a genetic laboratory (Supplementary Material, available as supplementary data at *Bioinformatics* online). All probands had DNA variants predicted to cause RNA alternative splicing (AS) of clinically suspected pathogenic genes (Table 5, available as supplementary data at *Bioinformatics* online). The control group consisted of 65 miscellaneous samples, including (i) unaffected parental samples of some probands and (ii) probands and parents not suitable for blood specimen-based aberrant expression analysis. All the samples were subjected to blood RNA-seq and sequenced on the same platform. Gene read counts were generated with an in-house pipeline.

To validate the splicing effect of the candidate variants, alternative splicing events proximal to the variants were manually inspected. Variants validated by aberrant splicing were considered pathogenic. Variants that were not validated were identified as VUS. After RNA-seq alternative splicing analysis, 30 cases were resolved, and 16 cases were undiagnosed. Suspected pathogenic genes in probands are considered as true outliers. Variant consequences were annotated via Ensembl VEP v115.2 LOFTEE plugin (Karczewski et al. 2020). Four hundred and fifty stop-gain predicted NMD triggering genes are considered as additional true outliers (Table 6, available as supplementary data at *Bioinformatics* online).

See the Supplementary Material (available as supplementary data at *Bioinformatics* online) for full details about dataset preprocessing.

#### 2.2.2 Semi-synthetic dataset for robustness tests

We analyzed the robustness of AXOLOTL with Kremer119, Pfibns154, and Pfibss269. Robustness refers to the ability of a method to consistently outperform other methods across multiple dataset settings, including varying sample sizes (10∼100) and percentages (4∼40%) of samples with aberrant gene expression. The raw dataset was randomly subsampled for the desired settings to obtain the simulated dataset. The performance of each setting was averaged across 10 simulations with different random seeds.

### 2.3 AXOLOTL method

AXOLOTL generates two features by feature engineering. One is *osg_p*, the log-transformed *P*-value from OutSingle. The other is *ord_p_devi*, a customized sample-specific deviation value derived from the log*P* and *z*-score of OUTRIDER. An overview of the method is shown in Fig. 1B. We next describe the implementation of feature construction and machine learning model.

#### 2.3.1 Constructing new features from OUTRIDER and OutSingle

##### 2.3.1.1 Feature 1. osg_p

We obtained the P-value statistic of OutSingle method. This statistic aims to quantify whether the gene expression level follows a negative binomial distribution from a statistical perspective (Salkovic et al. 2023). The P-value matrix is negative-logarithmically transformed and denoted as feature matrix *osg_p*.

##### 2.3.1.2 Feature 2. ord_p_devi

We quantified the extent to which a gene’s expression deviates from its coexpression partners’ collective pattern (hereafter “coexpression constraint”) as *ord_p_devi*. The newly customized feature *ord_p_devi* is based on OUTRIDER predictions.

###### (a) Standardization of gene expression matrix

The expression matrix $C_{0}$ of G genes (in row) and S samples (in column), where $C_{0}(g,s)$ represents a gene-sample pair. First, the matrix was preprocessed through OUTRIDER autoencoder normalization. The autoencoder learns latent low-dimensional encodings from raw expression profiles, and regenerates normalized gene expression profiles from latent encodings. The confounding factors (batch effect, technical variation, sequencing depth, etc.) in $C_{0}$ were controlled by the denoising autoencoder to create a normalized matrix C. The matrix C corresponds to the expected counts generated by OUTRIDER. Compared to the original count matrix $C_{0}$, the gene expression levels in C are more comparable across samples. The optimal latent encoding dimension of the autoencoder is searched within integers ranging from $\frac{1}{9}S∼\frac{1}{3}S$. Second, the *z* scores-based features were computed. The expression matrix C is standardized across all samples to construct the *z*-score matrix Z.

###### (b) Identification of gene coexpression partners

To determine the coexpression partners of each gene, we first computed Pearson’s correlation coefficient (PCC) matrix R for all pairs of G genes using the *z*-score matrix Z. The value $R(g,g_{i})$ represents the coexpression strength between focal gene g and another gene $g_{i}$. The closer *R*(*g*, *gi*) is to 1, the stronger the linear correlation between the two genes. For each g, the top p% of genes with the largest $R(g,g_{i})$ are defined as most strongly coexpressing partners, denoted as $G_{\mathrm{co}}(g)$. The optimal percentage p is searched in range of 0.05∼4.

###### (c) Measurement of deviation from coexpression constraints

We first obtained the adjusted P-value statistic of OUTRIDER method. The P-value matrix is negative-logarithmically transformed and denoted as feature matrix $P_{\mathrm{ord}}$. Then we obtained $P_{\mathrm{ord}}(g,s)$ of the focal gene g in sample s, then computed the average of $P_{\mathrm{ord}}(g_{i},s)$ of its coexpression partners ($g_{i}\in G_{\mathrm{co}}(g)$) in the same sample. The difference between $P_{\mathrm{ord}}(g,s)$ and the average of $P_{\mathrm{ord}}(g_{i},s)$ was denoted as *ord_p_devi* of (g,s).

#### 2.3.2 Outlier detection model

AXOLOTL employs the Local Outlier Factor (LOF) algorithm for aberrant gene expression detection. The unsupervised machine learning model uses two key features:

1. *osg_p* derived from OutSingle, and
2. *ord_p_devi* derived from OUTRIDER.

The LOF model is fitted independently to the data of each gene. For a specific gene, the input feature matrix is of dimension S×2, with samples as rows and the *osg_p* and *ord_p_devi* features as columns. The model’s goal is to identify samples with aberrant expression of the specific gene. It is iteratively fitted to each gene and assigns anomaly scores to all genes across all samples. Outputs are in the same shape as the expression matrix.

LOF estimates the local density of each sample by considering the distance between its *k*-nearest neighbors. Samples with substantially lower local density than other samples are classified as outliers, indicating aberrant gene expression. The optimal k is searched in range of 10∼30.

#### 2.3.3 Alternative models

Two alternative outlier detection models—Isolation Forest (IF) and One-Class Support Vector Machine (OC-SVM)—were selected to verify the suitability of LOF for AXOLOTL. To conduct this validation, we replaced LOF with IF or OC-SVM in the AXOLOTL and compared their performance.

### 2.4 Performance evaluation

AXOLOTL hyper-parameters are optimized on GTEx dataset. Evaluations were conducted on both GTEx datasets and rare disease datasets. Representative baseline models OUTRIDER (Brechtmann et al. 2018), ABEILLE (Labory et al. 2022), and OutSingle (Salkovic et al. 2023) are used in performance comparisons. Genes are ranked by anomality score. Ideally, aberrantly expressed genes rank top in this list to reduce genetic counselors’ manual check workload. Model performance was evaluated via the Precision-Recall (PR) curve, the area under the PR curve (AUPRC), and the top-k hit recall. The AUPRC places significant emphasis on the model’s ability to prioritize positives in imbalanced datasets. Top-k hit recall is the proportion of outlier genes among the top k genes per proband sample. To do this, genes were ranked within each sample and compute recall at predefined top-k threshold.

### 2.5 Model explanation by feature attribution

Feature attributions are analyzed by the model-agnostic explanation methods SHAP (SHapley Additive exPlanations; Lundberg and Lee 2017) and LIME (Local Interpretable Model-Agnostic Explanations; Ribeiro et al. 2016). Feature attributions indicate how much each feature contributed to AXOLOTL’s output for samples of a given dataset.

The rare disease datasets Pmuscle36, Kremer119, Pfibns154, and Pfibss269 were used. Gene-specific models of disease-causative and random genes were analyzed (Table 7, available as supplementary data at *Bioinformatics* online). For Pmuscle36, the gene-specific models of disease genes (COL6A1, COL6A2, and COL6A3) of COL6-RD, causative genes of other phenotypically similar diseases (COL6A6, CAPN3, DYSF, TTN, TCAP, and LMNA), and 100 other random genes were included.

### 2.6 Implementation

The LOF, IF, and OC-SVM models were implemented using scikit-learn v1.4.2 neighbors.LocalOutlierFactor, ensemble.IsolationForest and svm.OneClassSVM with default parameters unless otherwise specified.

The search space for hyperparameter tuning was set as follows: [10, 40] for LOF’s n_neighbors, [linear, poly, rbf] for OneClassSVM’s kernel, and [50, 200] for IsolationForest’s n_estimators. PCC was calculated with scipy.stats from scipy v1.13.1. Feature importance analysis of SHAP and LIME was performed with scikit-explain v0.1.4. The plots were drawn using Matplotlib and Seaborn in Python-3.12. OUTRIDER v1.24.0 and ABEILLE v1.0.0 were implemented on R-4.4 and R-4.0. RNA AS was visualized using an Integrative Genomics Viewer (Robinson et al. 2011).

## 3 Results

### 3.1 Overview of the AXOLOTL method

AXOLOTL detects aberrant gene expression using RNA-seq read count data (Methods and Fig. 1B). The input of AXOLOTL is solely an RNA-seq read count *N_gene*×*N_sample* matrix. The output of AXOLOTL is an anomaly score matrix with the same shape as the input. Two features *osg_p* and *ord_p_devi* were constructed to measure the deviation of gene expression from the coexpression constraint. Using these features, an unsupervised outlier detection model was fit to detect rare aberrant gene expression events.

Aberrant gene expression identification of each gene is an anomaly detection task. The workflow consists of three steps. First, we normalize the gene expression matrix by OUTRIDER’s autoencoder component. The normalized gene expression level was more comparable across samples than was the raw read count. Second, we constructed *ord_p_devi* feature capturing the deviation of a gene from its normal expression range. By comparing one gene across many samples, the feature *ord_p_devi* based on OUTRIDER p values are used to quantify how aberrant it is in each sample. For one gene, other genes among the top 0.1% largest Pearson’s correlation coefficient were defined as coexpression partners. The coexpression partner relationships serve as constraints on gene expression variations in normal samples. The negative log-transformed *P*-value difference between this gene and coexpression genes measure how much a gene deviates from its coexpression partners in the profile. The feature *osg_p* is the negative log-transformed OutSingle p value. Third, for each gene, we fit an unsupervised outlier detection model using a gene-specific matrix of *N_sample* rows and two columns. Genes were looped through iteratively to detect aberrant gene expression.

### 3.2 AXOLOTL detects healthy individuals’ gene expression outliers

**Figure 2:**
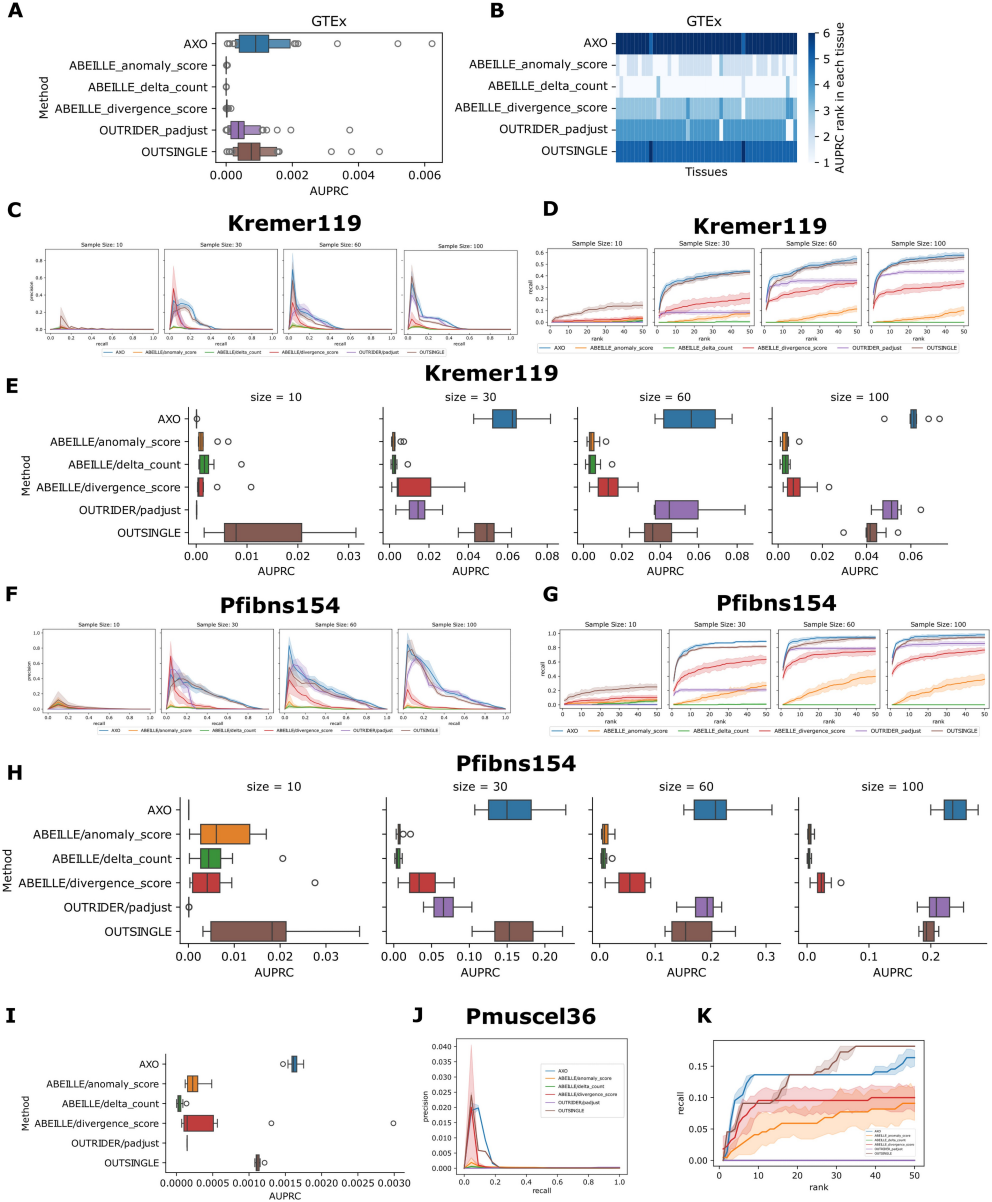
Performance comparison of AXOLOTL with other methods on public datasets. Performance on the GTEx dataset of 49 tissues from healthy donors: (A) boxplot of Area Under the PR curve (AUPRC); (B) heatmap of relative rank of AUPRC across tissues, sorted in ascending order. Performance on the Kremer119 dataset at various cohort sizes: (C) PR curve with 95% CI; (D) Top-1∼50 hits recall curve with 95% CI. (E) Boxplot of AUPRC. Performance on the Pfibns154 dataset at various cohort sizes: (F) PR curve with 95% CI; (G) Top-1∼50 hits recall curve with 95% CI. (H) boxplot of AUPRC. Performance on the Pmuscle36 dataset at simulated subset of 12 000 genes: (I) boxplot of AUPRC; (J) PR curve with 95% CI; (K)Top-1∼50 hits recall curve with 95% CI.

Healthy individuals in the GTEx database exhibit extreme outlier gene expression due to rare genetic variations (Ferraro et al. 2020). Since gene expression outliers are caused by rare genetic variations in the healthy population as well as rare disease patients, the gene expression dataset of GTEx donors is suitable for unbiased benchmarking of aberrant gene expression detection methods. From the GTEx database, we processed 49 GTEx tissue RNA-seq count matrices and utilized publicly available 1782 unique stop-gain NMD variants, resulting in an average of 584.7 outliers per tissue and 36.3 per individual (Fig. 1A). We split the outliers at an 8:2 ratio for training and testing datasets. To develop AXOLOTL, we used the training split to optimize hyperparameters, including the hidden dimension of OUTRIDER, coexpression threshold, and number of neighbors for the LOF model (Figs 1 and 2, available as supplementary data at *Bioinformatics* online). Based on these findings, we concluded that AXOLOTL is the optimal choice for its intended purpose. Two alternative outlier detection models—Isolation Forest (IF) and One-Class Support Vector Machine (OC-SVM)—were selected to verify the suitability of LOF for AXOLOTL (Fig. 2, available as supplementary data at *Bioinformatics* online). A full account of these analyses is provided in the Supplementary Methods (available as supplementary data at *Bioinformatics* online). When evaluated on the independent test split, AXOLOTL exhibited superior performance to three baseline models in detecting outliers in healthy individuals (Fig. 2A and B).

### 3.3 AXOLOTL outperforms existing methods on rare disease cohorts

We next evaluated the performance of the AXOLOTL in public rare disease cohorts.

**Figure 3:**
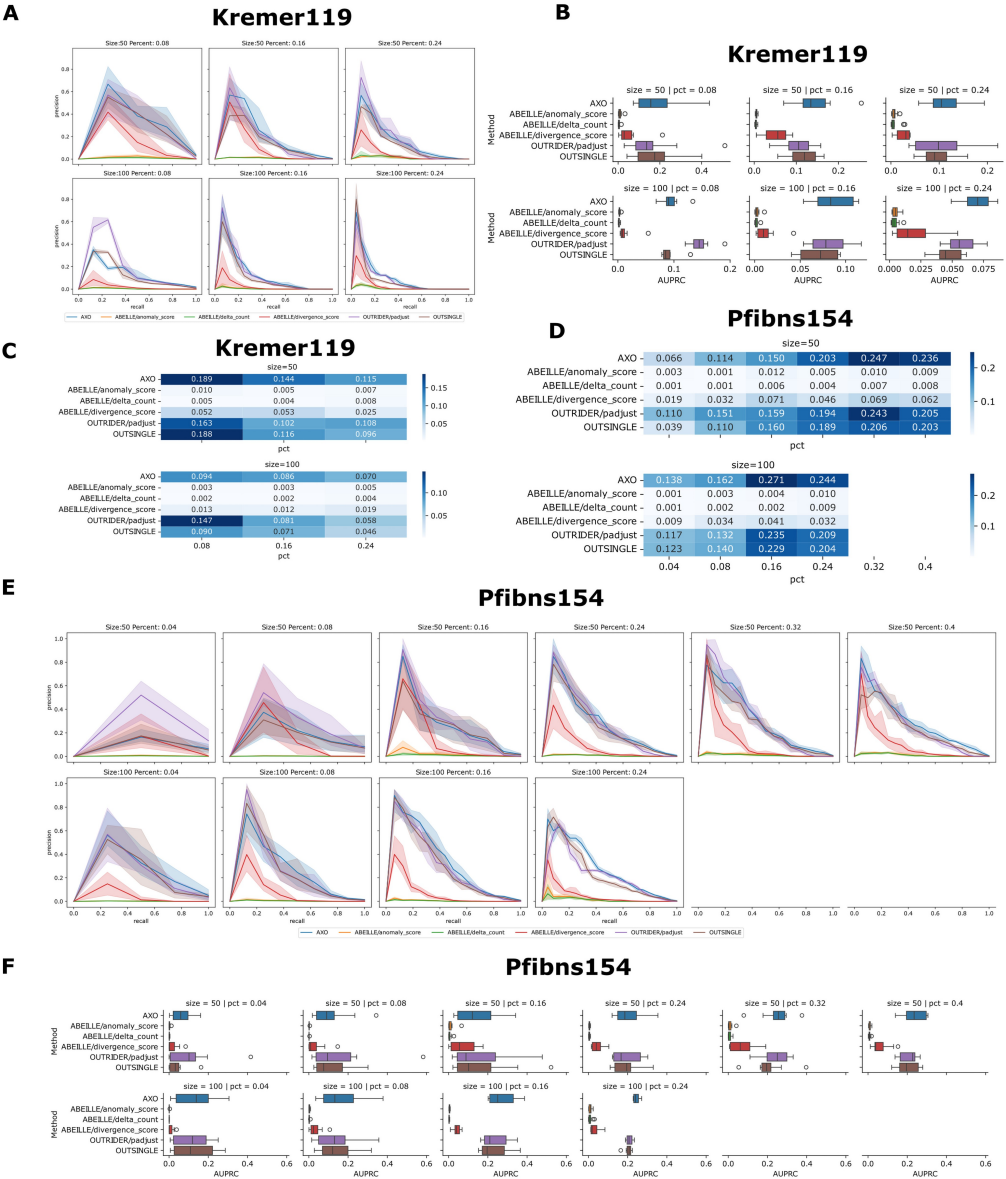
Robustness analysis of AXOLOTL on public datasets. Performance on Kremer119 dataset: (A) the Precision-recall (PR) curves with 95% CI; (B) boxplot of Area Under PR curve (AUPRC); (C) heatmap of average AUPRC values. Performance on Pfibns154 dataset: (E) the Precision-recall (PR) curves with 95% CI; (F) boxplot of Area Under PR curve (AUPRC); (D) heatmap of average AUPRC values. Simulations are conducted across sample sizes ranging from 50 to 100 and outlier sample percentages from 4% to 40%. Datasets were generated by random subsampling from the Kremer119 and Pfibns154 datasets, with 10 simulations performed for each cohort size and positive percentage.

The Kremer119 (Kremer et al. 2017), Pfibns154, and Pfibss269 (Yépez et al. 2022) datasets are cohorts of skin-derived fibroblasts from patients with mitochondrial disease or other Mendelian diseases. Twenty-two candidate NMD-associated outliers and six RNA-seq-validated outliers were designated as outliers in the Kremer119 dataset. A total of 49 patients were successfully diagnosed, with their causative genes confirmed via aberrant gene expression analysis (Yépez et al. 2022). These 49 sample–gene pairs were labeled as outliers in the Pfibns154 and Pfibss269 datasets. For a rigorous comparison, each dataset was subsampled to generate additional simulated datasets with varying sample sizes (Supplementary Methods, available as supplementary data at *Bioinformatics* online). The PR curves, top-k hit recall curves, and AUPRC values of different methods are presented for the Kremer119 dataset (Fig. 2C–E, Figs 3A and 5A, available as supplementary data at *Bioinformatics* online), Pfibns154 dataset (Fig. 2F–H, Figs 3B and 5B, available as supplementary data at *Bioinformatics* online), and Pfibss269 dataset (Figs 3C, E, F and 5C, available as supplementary data at *Bioinformatics* online).

AXOLOTL outperformed other methods at sample sizes of 30/60/100 in the Kremer119 dataset and 60/100 in the Pfibns154 dataset. OutSingle and AXOLOTL performed equivalently (both optimal) at a sample size of 30 in the Pfibns154 dataset. OUTRIDER ranked second at sample sizes of 60/100 in both the Kremer119 and Pfibns154 datasets. For the stranded Pfibss269 dataset, AXOLOTL was optimal at a sample size of 30, whereas OUTRIDER performed best at subsample sizes of 60/100. Notably, all reported outliers were originally identified by OUTRIDER using RNA-seq data, introducing a potential bias favoring OUTRIDER in these benchmarks.

AXOLOTL emerged as the most competitive method for non-stranded RNA-seq datasets—particularly at medium and large sample sizes. Despite this, OutSingle was the best at a sample size of 10 across all three datasets. The relative advantage of AXOLOTL demonstrates that the multivariate non-parametric approach successfully raises the upper limit of outlier detection performance relative to OUTRIDER and OutSingle. Collectively, these results confirm the superiority of AXOLOTL over other baseline methods for detecting aberrant gene expression in large cohorts.

Small cohorts of tens of patients’ RNA-seq data are more readily available than large cohorts. The performance of AXOLOTL on small datasets thus reflects its general reliability in diagnostic settings. The Pmuscle36 dataset comprises 22 patients with COL6-RDs and 14 controls (Guadagnin et al. 2021). Pathogenic variants in COL6A1, COL6A2, or COL6A3 were identified in all COL6-RD patients and validated via collagen immunostaining. Reduced COL6 protein and mRNA levels were observed in five patients. Known pathogenic genes were designated as true outliers in the simulated Pmuscle36 dataset through gene subsampling (Methods). We found that AXOLOTL outperformed other methods in terms of PR curves and AUPRC (Fig. 2I and J and Fig. 3D, available as supplementary data at *Bioinformatics* online). AXOLOTL detected more known outliers among the top 10 most aberrantly expressed genes in patients, while OutSingle did so among the top 30–50 (Fig. 2K).

Taken together, AXOLOTL outperforms other baseline methods in detecting expression outliers in representative rare disease datasets.

### 3.4 AXOLOTL is robust across various dataset compositions

To further evaluate the robustness of our AXOLOTL method, we generated a series of simulated datasets from Kremer119, Pfibns154, and Pfibss269 to represent more diverse cohort compositions (Methods). Given that these disease cohorts contain 10%–20% known outlier samples, we simulated datasets with even lower or higher proportions of positive samples at fixed cohort sizes to assess the performance robustness of AXOLOTL. We thus benchmarked AXOLOTL across positive sample proportions of 4%–40% and cohort sizes of 50/100.

**Figure 4:**
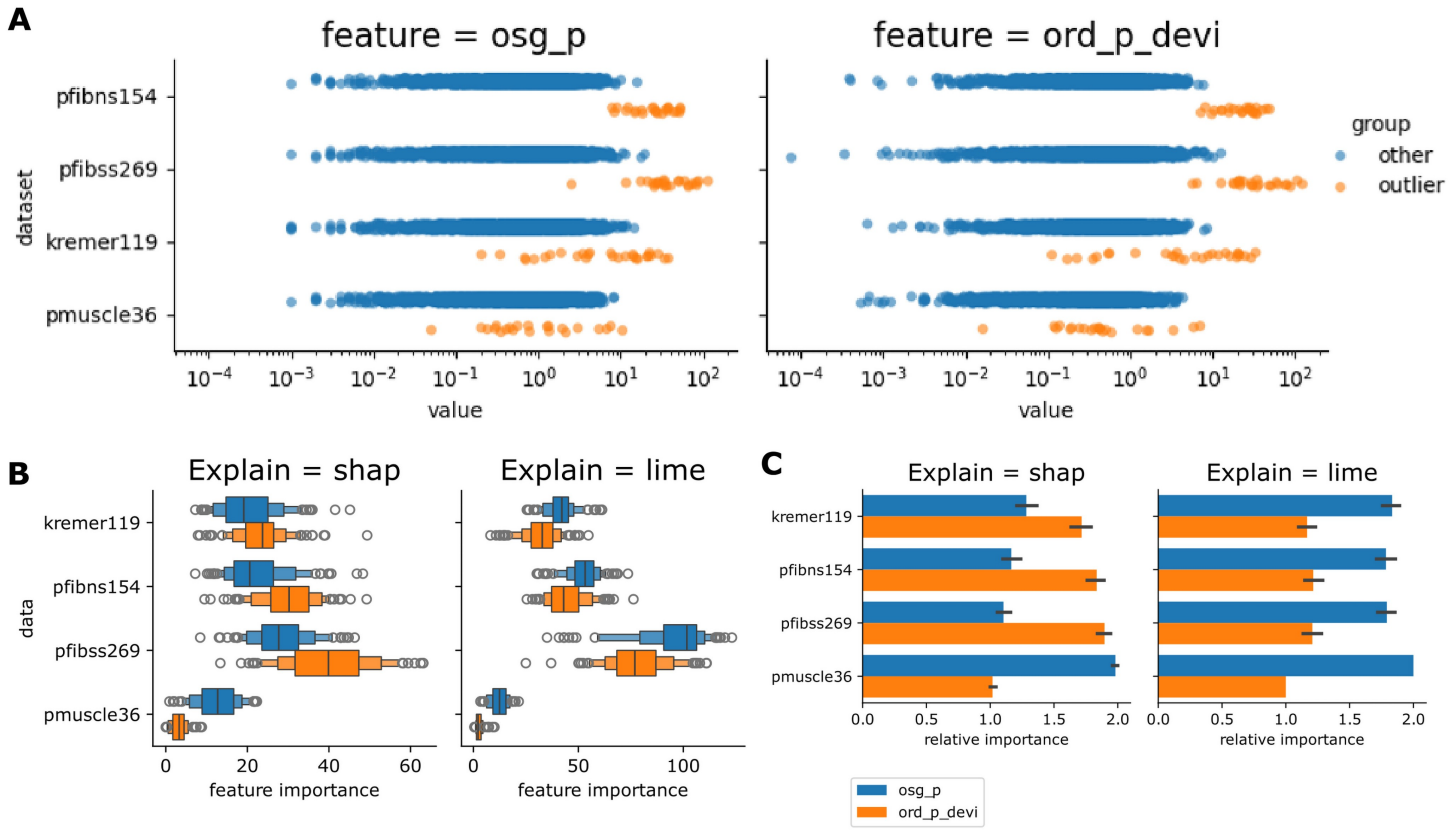
Model interpretation based on feature value distribution and importance. (A) Feature value distributions across four rare disease datasets. Outlier gene-sample pairs and a subset of randomly selected gene-sample pairs are visualized. (B) Feature importance analysis on rare disease datasets using SHAP and LIME methods. Boxplots display the feature attribution scores of gene-specific outlier detection models for disease-causative genes and a set of random genes. (C) Barplots show the relative importance of individual features.

We first assessed performance using AUPRC values. PR curves, top-k hit recall curves, and AUPRC values for different methods are presented for the Kremer119 dataset (Fig. 3A–C, Figs 4A and 6A, available as supplementary data at *Bioinformatics* online), Pfibns154 dataset (Fig. 3D–F, Figs 4B and 6B, available as supplementary data at *Bioinformatics* online), and Pfibss269 dataset (Figs 4C–F and 6C, available as supplementary data at *Bioinformatics* online). For the Kremer119 dataset, AXOLOTL outperformed other methods at positive sample proportions of 16%–24% across both cohort sizes of 50 and 100. At a sample size of 50 and positive sample proportion of 8%, AXOLOTL performed equivalently to OutSingle. In contrast, OUTRIDER achieved the best performance at a sample size of 100 and positive sample proportion of 8%. For the Pfibns154 dataset, AXOLOTL performed the best at a sample size of 50 (proportions of 24% and 40%) and a sample size of 100 (proportions of 16% and 24%); OUTRIDER, OutSingle, and AXOLOTL performed equivalently under all other settings. For the Pfibss269 dataset, OUTRIDER exhibited the best performance across all tested settings.

These results further confirm that AXOLOTL is the most competitive method for non-stranded RNA-seq datasets, and demonstrate its robustness to variations in cohort size and positive sample proportion.

### 3.5 Feature distribution and model explanation

To facilitate a comprehensive understanding of the relative importance of the two customized features, we next interpreted the AXOLOTL model via feature attribution-based explanations. Feature attributions reflect the relative importance of each feature in explaining AXOLOTL’s output. For every feature, aberrantly expressed gene-sample pairs exhibited larger absolute attribution values than normal pairs (Fig. 4A).

We assessed feature importance using two model-agnostic methods: the game theory-based SHAP and the linear model-based LIME. These analyses were performed on the Pmuscle36, Kremer119, Pfibns154, and Pfibss269 datasets (Fig. 4B and C). SHAP and LIME attribution values showed wide variability across the selected sample–gene pairs (Fig. 4B); however, the relative ranking of feature attributions remained highly stable for each sample–gene pair (Fig. 4C). Feature importance derived from SHAP and LIME values indicated that *osg_p* and *ord_p_devi* are equally important overall. Interestingly, *osg_p* contributed more significantly in the Pmuscle36 dataset. Collectively, these results suggest that coexpression features provide valuable information for outlier detection in large cohorts.

### 3.6 Real case study

We next aimed to explore its potential in facilitating the filtering and interpretation of results derived from a blood-based RNA-seq cohort of rare disease samples.

We applied the AXOLOTL method to the in-house Ly111 dataset to identify aberrant gene expression from blood RNA-seq profiles. This dataset comprises 65 control samples and 46 probands predominantly diagnosed with neurological disorders (Table 5, available as supplementary data at *Bioinformatics* online). Most probands presented phenotypes linked to causal DNA variants that SpliceAI predicted to impair splicing (Supplementary Methods, available as supplementary data at *Bioinformatics* online). We first manually inspected the genomic regions flanking these variants for evidence of aberrant splicing events. Integrating DNA-level pathogenicity evidence and RNA alternative splicing (AS) signatures adjacent to these variants, we resolved 30 cases, with 16 remaining undiagnosed. Subsequently, we used AXOLOTL to independently detect aberrant expression events, and we sought supporting molecular evidence for both diagnosed and undiagnosed cases. Additionally, stop-gain variants predicted to trigger nonsense-mediated decay (NMD) were included as benchmark data (Table 6, available as supplementary data at *Bioinformatics* online).

**Figure 5:**
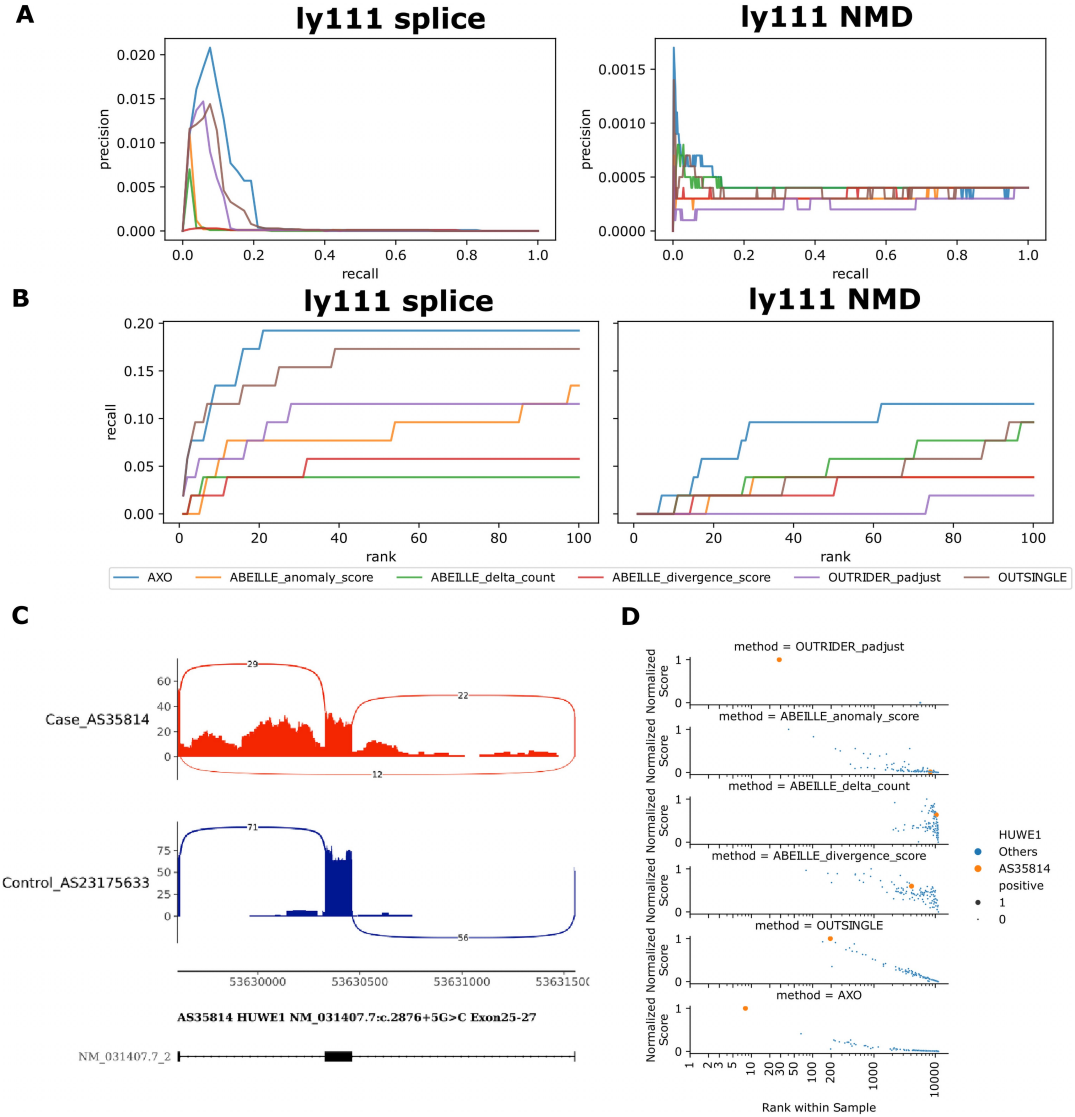
Performance comparison of AXOLOTL against alternative methods on blood RNA-seq samples from the rare disease dataset ly111. (A) Precision-recall (PR) curves with aberrant splicing as potential outliers (left) and predicted nonsense-mediated decay (NMD) as potential outliers (right). (B) Recall curves for top-1 to top-50 hits, using aberrant splicing (left) and predicted NMD (right) as potential outliers. (C) Alternative splicing profile of the HUWE1 gene in the proband, compared with a representative control sample. (D) Comparison of HUWE1 gene rankings and normalized aberration scores across different methods; the horizontal axis represents log_2_-transformed rankings.

The aberrant genes of these cases and predicted NMD genes were ranked higher by AXOLOTL than by other methods (Fig. 5A and B). The advantages in giving high rank for causal genes of AXOLOTL method are illustrated with one case AS35814. In the male proband (HP: 0001263: global developmental delay; HP: 0001274: agenesis of corpus callosum; HP: 0001513: obesity) with dominant X-linked intellectual developmental disorder, HUWE1 variant (chrX: 53630324, c.2876 + 5G>C) caused alternative splicing via exon skipping (Fig. 5C). Aberrant ranking of the causal gene HUWE1 in the proband was 8 for AXOLOTL, which was higher than 194, 4000, and 28 for OutSingle, ABEILLE, and OUTRIDER, respectively (Fig. 5D). The example shows that AXOLOTL can prioritize aberrant expression events that are less distinguishable via other methods.

## 4 Conclusion and discussion

AXOLOTL provides a powerful approach for prioritizing aberrant gene expression. This study validates the effectiveness of AXOLOTL, as evidenced by its successful recovery of known cases from publicly available datasets as well as our in-house dataset. In general, AXOLOTL assigned higher rank to genes exhibiting aberrant expression in both the healthy and rare disease cohorts. Crucially, AXOLOTL exhibits robust performance and high sensitivity even in the small Pmuscle36 cohort, making it an attractive tool for a broad range of users. The performance gains of AXOLOTL are modest in certain contexts (Fig. 2A and I). Additionally, OUTRIDER outperforms it in the Pfibss269 stranded dataset, and OutSingle excels at very small sample sizes. We recognize that no single method is universally optimal. Regarding computational cost and scalability, runtime and peak memory usage of AXOLOTL increase linearly with sample size (Fig. 7, available as supplementary data at *Bioinformatics* online).

The approach of AXOLOTL to extend post-OUTRIDER/OutSingle features is informative and significantly enhances the accuracy of outlier detection. We also proposed the use of coexpression constraints as a metric for gene correlation. AXOLOTL leverages sample-specific variability to detect gene aberrations. Model explainability analysis suggests that the two features provide complementary insights.

In the performance evaluation of AXOLOTL, no artificial corruption was employed. This approach contrasts with prior studies, which incorporated read count fold-change corruption into random sample–gene pairs while leaving the remaining genes unchanged (Brechtmann et al. 2018, Labory et al. 2022). While the introduction of artificial corruptions can facilitate method development, fold-change corruption does not accurately recapitulate any rare disease. In contrast, pathogenic genes result in transcriptional changes across many genes. Given that aberrant gene expression events naturally occur in GTEx individuals (Ferraro et al. 2020), the NMD-triggering events in GTEx are suitable as benchmark outlier data for performance evaluation.

The AXOLOTL score can be incorporated as RNA-seq-based supporting evidence for the prioritization of pathogenic genes—a key challenge in rare disease diagnosis. In diagnostic pipelines, when DNA sequencing identifies a candidate variant, AXOLOTL scores are queried to assess RNA-level functional impact, providing ACMG—aligned PS3/BS3 evidence for classifying VUS.

This study has three limitations. First, all methods demonstrated low precision beyond a recall of 0.8, indicating that defining a clean set of true outliers is challenging. In such cases, pathogenic genes with splicing or protein function defects may not exhibit extreme gene expression levels, which limits clinical utility in its current form. Second, the AXOLOTL method is designed to improve the accuracy of detecting aberrations in probands rather than directly evaluating pathogenicity. Third, we acknowledge that while NMD variants enhance the orthogonality of our study, not all NMD variants result in detectable aberrant expression, and certain pathogenic mechanisms (e.g. missense, regulatory variants) do not trigger NMD. This means the ground truth used in our evaluation remains imperfect.

In summary, AXOLOTL enhances the detection of aberrant gene expression, demonstrating its advantages in real-world cohort studies. By highlighting the importance of coexpression as an essential biological constraint, we hope this work will ultimately empower data-driven rare disease diagnosis and inspire methodological innovations from new perspectives.

## Supplementary Material

btag255_Supplementary_Data

## Data Availability

The sequencing data of the ly111 dataset have not been deposited in a public repository to protect individual confidentiality. Our ethics approval and consent agreements allow us to share nonidentifiable patient data and gene expression count matrix only. They are available for academic research at https://doi.org/10.5281/zenodo.10408481. The code is available at https://doi.org/10.5281/zenodo.17940844. Supporting data of other datasets were downloaded from original publications.

## Declarations

Consent for publication: We have obtained consent to publish from the participants to report individual patient data.
