# Supplementary material for: AXOLOTL: an accurate method for detecting aberrant gene expression in rare diseases using coexpression constraints
Source: Bioinformatics. 2026 May 4;42(5):btag255. doi: 10.1093/bioinformatics/btag255 (PMC13198384; doi:10.1093/bioinformatics/btag255)
Supplement: btag255_Supplementary_Data [file btag255_supplementary_data.zip › Supplemental Methods_v3_MinorRev.docx]

# AXOLOTL: an accurate method for detecting aberrant gene expression in rare diseases using co-expression constraints

## Supplemental Methods

### Raw dataset preprocessing

#### GTEx cohort

We used three files from GTEx Portal to define expression outliers in GTEx datasets.

File1: GTEx_Analysis_v8_Annotations_SampleAttributesDS.txt

File2: GTEx_Analysis_2017-06-05_v8_RNASeQCv1.1.9_gene_reads.gct.gz

File3: gtexV8.eOutlier.stats.globalOutliers.removed.txt.gz

We used one file from [1] to define predicted-NMD outliers in GTEx samples.

File4: 1-s2.0-S0002929721002329-mmc4.xlsx (The paper[1]’s Table S3 “Complete annotations and NMD predictions for all stop gain variants analyzed in this study“ )

**Find high-quality samples of these tissues**. File1 contains sample ids and sample annotations of all tissues. We kept the high quality samples with SMRIN greater than 5.6. We use 'SAMPID’ and ‘SUBJID’ as sample ids.

**Prepare read counts matrices**. File2 are RNA-seq read counts (v8) provided by GTEx portal. The dataset consisted of 17,382 transcription profiles from 54 healthy tissues of 948 donors. Duplicate experiments of same samples are merged by keeping maximum count value. Multiple transcripts of the same gene were merged. RNA-seq profiles of 49 tissues were separated by tissue. For each tissue, we retained genes with ≥10 reads across all samples. Each profile contains expression levels of >10,000 genes.

**Define true expression outliers**.

We utilized publicly available stop-gain NMD variants from [1] and processed File 4 and 49 GTEx tissue RNA-seq count matrices. We identified 1,782 unique NMD-harboring genes in total, resulting in an average of 584.7 outliers per tissue and 36.3 per individual. markedly increasing the true outlier.

File3 describes expression outliers in individual donors using z-score stat [2]. These outliers are globally aberrant expressing genes in multi-tissues; thus the outliers are caused by special genetic background of healthy donors. We checked z-score amplitude of NMD related genes, and found the expression outliers enriched in NMD genes.

GTEx NMD true outliers are provided as **Supplemental Table S1.**

#### Rare disease cohort 1 - Kremer119 Dataset

The outlier dataset included two components: (1) 22 candidate NMD-associated outliers (2) six RNA-seq-validated outliers from prior OUTRIDER [3] and ABEILLE [4] studies.

First, potential NMD expression outliers were derived from the sample annotation table (S3_41467_2017_BFncomms15824_MOESM392_ESM.txt) of [5] as our team lacks authorization for raw data. Specifically, variants were annotated as "nmd" if the VARIANT_EFFECT field contained "stop" or "frame-shift" terms. We then extracted entries with this "nmd" predicted effect, retaining information on diagnostic genes, variant effects, and RNA defects. These formed candidate NMD-associated expression outliers linked to diagnostic genes and sample metadata. Second, we included six RNA-seq-validated outliers previously used in the OUTRIDER and ABEILLE studies. In total, the Kremer119 dataset contained 28 outliers. All outliers are provided as **Supplemental Table S2**.

#### Rare disease cohort 2 - stranded (pfib_ss269) and non-stranded (pfib_ns154)

Yépez et al. dataset, the largest publicly available rare disease RNA-seq cohort data to date, is a dataset of skin-derived fibroblasts of 423 patients suffering from mitochondrial disease or other Mendelian diseases [6].

**Prepare read counts matrices**. The RNA-seq read counts were downloaded as two parts (Zenodo repository record 4646823 and 4646827), stranded specific table (fib_ss--hg19--gencode34/geneCounts.tsv.gz) and non-strand specific table (fib_ns--hg19--gencode34/geneCounts.tsv.gz). The two tables were processed separately. The genes with read count > 10 in all samples were retained.

**Define true expression outliers**. Supplemental Tables of paper [6] (Table S2. Summary of cases diagnosed via RNA-seq and Table S4. Summary of WES-diagnosed cases with an RNA-defect) were manually processed. 49 aberrant expression events (labeled as ‘AE’ in the original publication) were compiled and used as true outliers of the dataset (**Supplemental Table S3**). The positives were reported in RNA-seq aberrant expression analysis and also confirmed by WES/WGS.

**Simulation by subsampling**. Furthermore, raw datasets are used to analyze the robustness of AXOLOTL at settings of different sample size and different percent of positive patients. We set the sample size to 10~100 and the positive sample percentage to 0.04~0.40. To obtain simulated datasets, we randomly sampled 10 times for each (sample-size, percentage) settings. Simulated datasets were analyzed by AXOLOTL and baseline methods.

#### Rare disease cohort 3 – pmuscle_36

Pmuscle_36 is a RNA-seq dataset of skeletal muscle biopsies of 22 COL6-RD patients and 14 age-matched controls [7]. COL6-RD, a congenital muscular dystrophy with muscle weakness and joint defects, involves COL6 microfibril loss or dysfunction in the muscle extracellular matrix. The molecular mechanism of COL6-RD is loss of function or dysfunction of COL6 microfibrils in the muscle extracellular matrix. COL6-RD can be caused by pathogenic variants in collagen VI genes (COL6A1, COL6A2, COL6A3). Pathogenic variants in COL6A genes were found in 22 patients. Immunostaining assays indicated that COL6A proteins were aberrantly low expressed in 5 patients and mis-localized in the other 17 patients. (**Supplemental Table S4**). Gene read counts were produced using our in-house pipeline described in [8] from RNA-seq raw data GSE103975.

#### Rare disease cohort 4 – ly111

**Sample collection and variant pathogenicity assessment**

We established stringent inclusion criteria for patients diagnosed with rare diseases (**Supplemental Table S5**). Firstly, the proband's phenotype was utilized as a robust predictor of a monogenic rare disease. Secondly, variants on suspected pathogenic genes were identified through WES/WGS, or Sanger sequencing results of the proband and parents, with annotation to the HGVS-nomenclature and classification according to the ACMG guideline. Thirdly, the proband presented uncertain significance (VUS), likely pathogenic (LP), or pathogenic (P) DNA variants with an alteration in splice effect (SpliceAI v1.3 score > 0.2) in the disease gene.

The investigation of variants of VUS necessitates the application of RNA-seq analysis to validate their predicted splicing defects. Our approach utilized blood RNA-seq on both resolved and unresolved samples. The pre- and post-RNA-seq splicing analysis classifications were delineated in columns ‘ACMG_DNA’ and ‘ACMG_RNAseq’. The former corresponds to the standard analysis with SpliceAI-predicted splicing effects, while the latter pertains to the variant classification based on WES/WGS and manually verified in Integrative Genomics Viewer (IGV). Splicing defects observed in RNA-seq data were considered as supporting evidence. The identification of alternative splicing events proximal to the VUS variant was instrumental in upgrading some variants to LP/P classification. Conversely, VUSs were maintained if no abnormal splicing findings were observed. Notably, through abnormal splicing analysis, we elevated the pathogenicity of selected VUS variants to LP/P and identified candidate genes in these cases.

**Sample group definition**

The classification of variants in the 'ACMG-RNAseq' column represents the definitive basis for our conclusive diagnosis in each case: any likely pathogenic (LP) or pathogenic (P) variant is diagnosed, while VUS remains undiagnosed. Consequently, cases were categorized into diagnosed and undiagnosed groups based on this classification. Additionally, to expand the size of the ly_111 cohort, we incorporated additional samples to serve as a control group.

Diagnosed group (n=28). The samples have alternative splicing related LP/P level alleles, which are:

- 9 heterozygous cases affected by AD disorders,
- 5 homozygous cases affected by AR disorders,
- 12 compound heterozygous cases affected by AR disorders,
- 2 hemizygous males affected by X-linked disorders.

Undiagnosed group(n=18): the samples have only VUS level alleles, which are:

- 5 heterozygous cases affected by AD disorders,
- 4 heterozygous cases affected by AR disorders,
- 1 homozygous case affected by AR disorders,
- 6 compound heterozygous cases affected by AR disorders,
- 2 Heterozygous females affected by X-linked disorders

Control group 1: unaffected adult individuals in probands’ family (n=43).

Control group 2: Miscellaneous samples (n=22):

- probands affected by genes lowly expressed in blood;
- probands who might have parental mosaic variants;
- probands of copy number variant, covering a few exons of one gene or multiple genes.
- probands and carriers of the thalassemia HBB variants. HBB gene is highly expressed in blood and is well-suited for the assessment of alternative splicing. However, the measurement of its expression level poses a challenge due to the considerable variability in HBB mRNA levels within blood samples.

**RNA-seq, read alignment, quality control and splicing analysis**

All samples were subjected to Whole Blood RNA-seq protocol and sequenced on Illumina NovaSeq 6000 platform with 150 bp paired-end reads. Total RNA was isolated with TRIzol-based RNA extraction. Quality of RNA was assessed by determination of the RNA integrity number (RIN) with a Bioanalyzer (Agilent). Next, polyadenylated RNA (mainly mRNA) was enriched with theNEBNext Poly(A) mRNA Magnetic Isolation Module (NEB, USA) followed by fragmentation, cDNA synthesis and library construction with the NEBNext Ultra™ II RNA Library Prep Kit for Illumina (NEB, USA). A minimum of 50 million reads were generated per sample. FASTQ files were processed with an inhouse pipeline. Raw reads were cleaned with fastp (v0.20.0), mapped with HiSat2 (v.2.1.0) to the GRCh38 GENCODE genome assembly and Gencode v41 as transcriptome reference. SNPs and indels were called using the GATK short variant discovery recommended pipeline with GATK (4.1.3.0) and STAR (2.5.3). Alternative splicing events were called by rMATS (v4.1.2) and visualized in the Integrated Genome Viewer (IGV; v2.16.2) and ggsashimi (v1.0.0).

**True outlier labelling for AXOLOTL performance evaluation**

Since our goal is evaluating the performance advantage of the AXOLOTL method, independent of the alternative splicing results, our focus centers on the validation of pathogenic genes through aberrant expression analysis. In this context, suspected pathogenic genes in probands are considered as true outliers. Variant consequences were annotated using Ensembl VEP v115.2 with the LOFTEE plugin[9]. Variants flagged as 'HC' (High Confidence) and 'stop-gain' were considered predicted to trigger nonsense-mediated decay (NMD). We use stop-gain NMD variants as true outliers as well. NMD variants resulted in 450 additional outliers. All outliers are provided as **Supplemental Table 13 and 14**. The aberrant rankings of these true outliers assigned by AXOLOTL and other methods is compared.

### Implementation

Feature importance analysis of SHAP and LIME are implemented by scikit-explain v0.1.4. Briefly, *skexplain.ExplainToolkit* was used to wrap the model LOF and feature matrix as *explainer* object. Then the SHAP and LIME results were retrieved from *explainer.local_attributions.* Next, by convert ‘shap_values__LOF’ and ‘lime_values__LOF’ values to importance scores for plotting purpose by *to_skexplain_importance*.

OUTRIDER v1.24.0 was installed on R-4.4.3 locally using R devtools command devtools::install_github( 'gagneurlab/OUTRIDER', dependencies=TRUE ). Default parameters of OUTRIDER function is used.

ABEILLE v1.0.0 were implemented by R packages running on R-4.0.0. It is installed using R devtools command devtools::install_github("UCA-MSI/ABEILLE", dependencies=TRUE).

### Optimization of hyperparameters with GTEx dataset

**Prepare feature ord_p_devi**

First, find the optimal hidden dimension of OUTRIDER. We test a range of dimention across ranging from 1/9 to 1/3 of the sample size. The result on training split suggest 1/3 is the best setting (Figure S1C-E).

Second, symantanouly find the best pearson’s correlation coefficience threshold for coexpression partners and n_neighbors value for LOF model. The range of coexpressed genes is the top 0%–4% when we calculate feature *ord_p_devi*. Neighbor sizes in the Local Outlier Factor (LOF) model is in the range of 10–40 neighbors. The result on training split (Figure S1F-I) suggest 0.1% and 20 neighbors is the best.

**Validate suitability of LOF in our method**

Replacement of the Local Outlier Factor (LOF) model in AXOLOTL with Isolation Forest(IF) or One-Class SVM(OC-SVM). The first alternative model is IF. It builds a recursive partitioning tree structure by randomly selecting one feature and then randomly splitting by the selected feature. The number of splits required to isolate a given sample from the root node is the sample’s path length. An extreme short average path length implies the sample is likely an outlier.

The second comparative model is OC-SVM. It assumes that normal samples are mainly from one class with smooth boundaries determined using input data. Samples that lie far outside the normal class boundary are classified as outliers.

Modified versions of AXOLOTL were constructed using Isolation Forest with varying n_estimators (I50–I200 denote 50 to 200 estimators) and One-Class SVM with different kernel functions (Or, Op, Ol represent the rbf, poly, and linear kernels, respectively). The results on training split (Figure S2A-D) suggest LOF is the best. Although IF is another optimal model, the computing time for LOF is much shorter than IF. We redraw the performance comparision with the best setting for each base model(Figure S2E-F).

### Optimization of Hyperparameters with the GTEx Dataset

**Preparation of the ord_p_devi Feature**

First, we determined the optimal hidden dimension of OUTRIDER by testing values ranging from 1/9 to 1/3 of the sample size. Results from the training split indicated that 1/3 of the sample size was the optimal setting (Figure S1C–E). Second, we simultaneously identified the optimal Pearson’s correlation coefficient threshold for coexpression partners and the optimal n_neighbors value for the Local Outlier Factor (LOF) model. For the ord_p_devi feature calculation, coexpressed genes were restricted to the top 0%–4% of pairwise correlations. The n_neighbors parameter of the LOF model was tested across 10–40 neighbors. Training split results (Figure S1F–I) showed that the optimal threshold for coexpression partners was 0.1%, with 20 neighbors for the LOF model.

**Validation of LOF Suitability for AXOLOTL**

To validate the LOF model, we replaced it in AXOLOTL with two alternative outlier detection methods: Isolation Forest (IF) and One-Class SVM. Modified AXOLOTL variants were constructed using IF with n_estimators ranging from 50 to 200 (denoted I50–I200) and One-Class SVM with three kernel functions—radial basis function (rbf, Or), polynomial (poly, Op), and linear (Ol). Training split results (Figure S2A–D) confirmed that the original LOF model outperformed both alternatives. While IF was a suboptimal alternative, LOF exhibited lower computational cost. We further plotted the performance comparison using the optimal parameter settings for each base model (Figure S2E–F).

### References

1. Teran NA, Nachun DC, Eulalio T, et al. Nonsense-mediated decay is highly stable across individuals and tissues. Am. J. Hum. Genet. 2021; 108:1401–1408

2. Ferraro NM, Strober BJ, Einson J, et al. Transcriptomic signatures across human tissues identify functional rare genetic variation. Science 2020; 369:eaaz5900

3. Brechtmann F, Mertes C, Matusevičiūtė A, et al. OUTRIDER: A Statistical Method for Detecting Aberrantly Expressed Genes in RNA Sequencing Data. Am. J. Hum. Genet. 2018; 103:907–917

4. Labory J, Le Bideau G, Pratella D, et al. ABEILLE: a novel method for ABerrant Expression Identification empLoying machine LEarning from RNA-sequencing data. Bioinforma. Oxf. Engl. 2022; 38:4754–4761

5. Kremer LS, Bader DM, Mertes C, et al. Genetic diagnosis of Mendelian disorders via RNA sequencing. Nat. Commun. 2017; 8:15824

6. Yépez VA, Gusic M, Kopajtich R, et al. Clinical implementation of RNA sequencing for Mendelian disease diagnostics. Genome Med. 2022; 14:38

7. Guadagnin E, Mohassel P, Johnson KR, et al. Transcriptome analysis of collagen VI‐related muscular dystrophy muscle biopsies. Ann. Clin. Transl. Neurol. 2021; 8:2184–2198

8. Xu W, He H, Guo Z, et al. Evaluation of machine learning models on protein level inference from prioritized RNA features. Brief. Bioinform. 2022; 23:bbac091

9. Karczewski KJ, Francioli LC, Tiao G, et al. The mutational constraint spectrum quantified from variation in 141,456 humans. Nature 2020; 581:434–443
