# Supplementary material for: AXOLOTL: an accurate method for detecting aberrant gene expression in rare diseases using coexpression constraints
Source: Bioinformatics. 2026 May 4;42(5):btag255. doi: 10.1093/bioinformatics/btag255 (PMC13198384; doi:10.1093/bioinformatics/btag255)
Supplement: btag255_Supplementary_Data [file btag255_supplementary_data.zip › SupplementalFigures_MinorRev.docx]

**
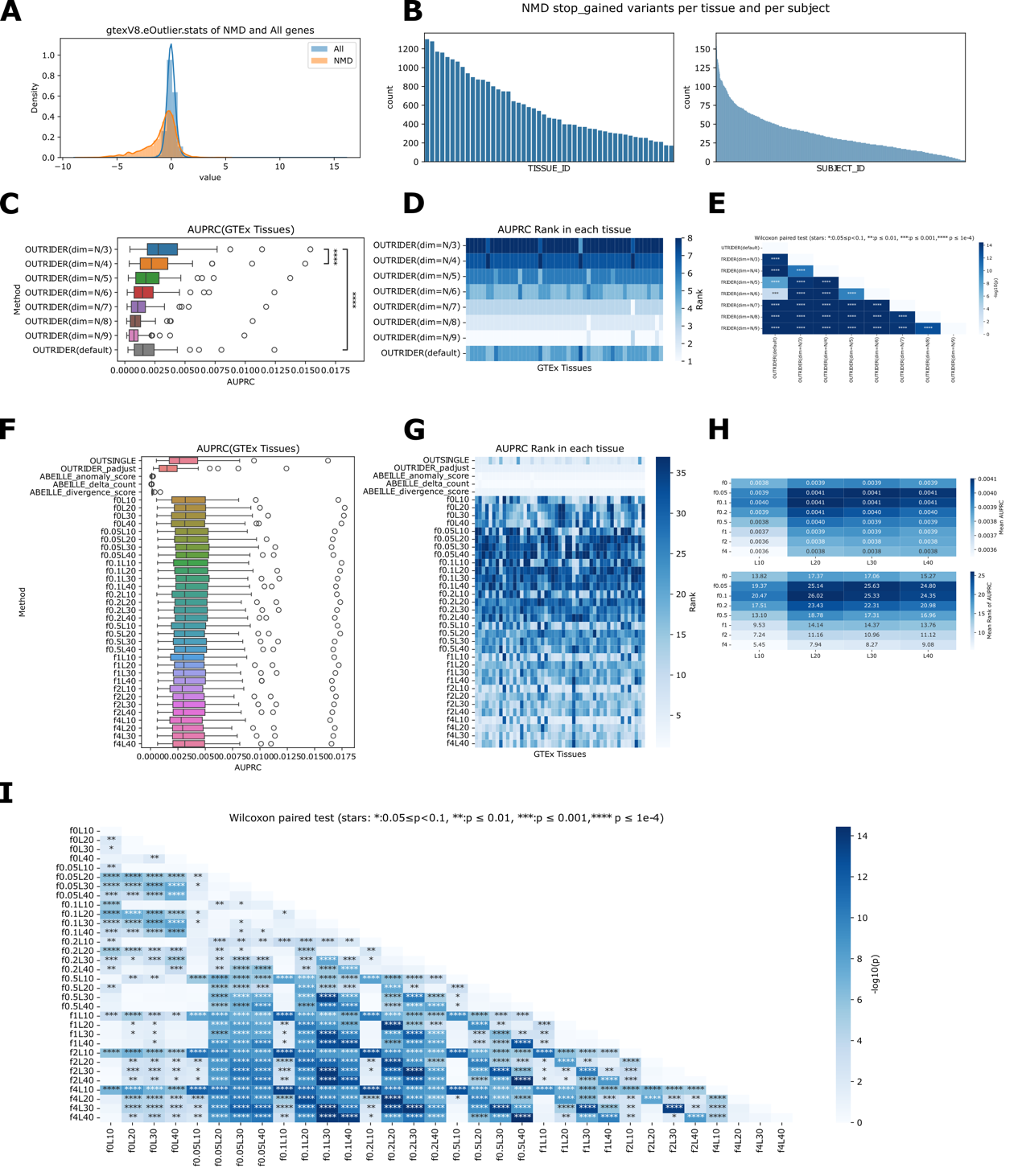
**

**Figure S1.** Performance optimization during the development of AXOLOTL and comparison with alternative methods on the GTEx dataset of 49 tissues.

(A) Expression outlier metric values (*eOutlier.stats*) for stop-gain predicted nonsense-mediated decay (NMD) genes. (B) Number of NMD genes per tissue and per individual. Performance of AXOLOTL’s input score 1 (OUTRIDER-padjust) across input dimensions ranging from 1/9 to 1/3 of the sample size: (C) Area under the precision-recall curve (AUPRC); (D) Heatmap of AUPRC relative ranks across tissues, sorted in ascending order; (E) *p*-values from Wilcoxon paired tests between different dimensions. Performance of AXOLOTL when using distinct thresholds (f0–f4 denote the top 0%–4% of coexpressed genes) to calculate feature *ord_p_devi* and varying neighbor sizes in the Local Outlier Factor (LOF) model (L10–L40 denote 10–40 neighbors): (F) AUPRC; (G) Heatmap of AUPRC relative ranks across tissues, sorted in ascending order; (H) Heatmap of average AUPRC across all threshold-neighbor combinations; (I) *p*-values from Wilcoxon tests between different parameter settings.

**
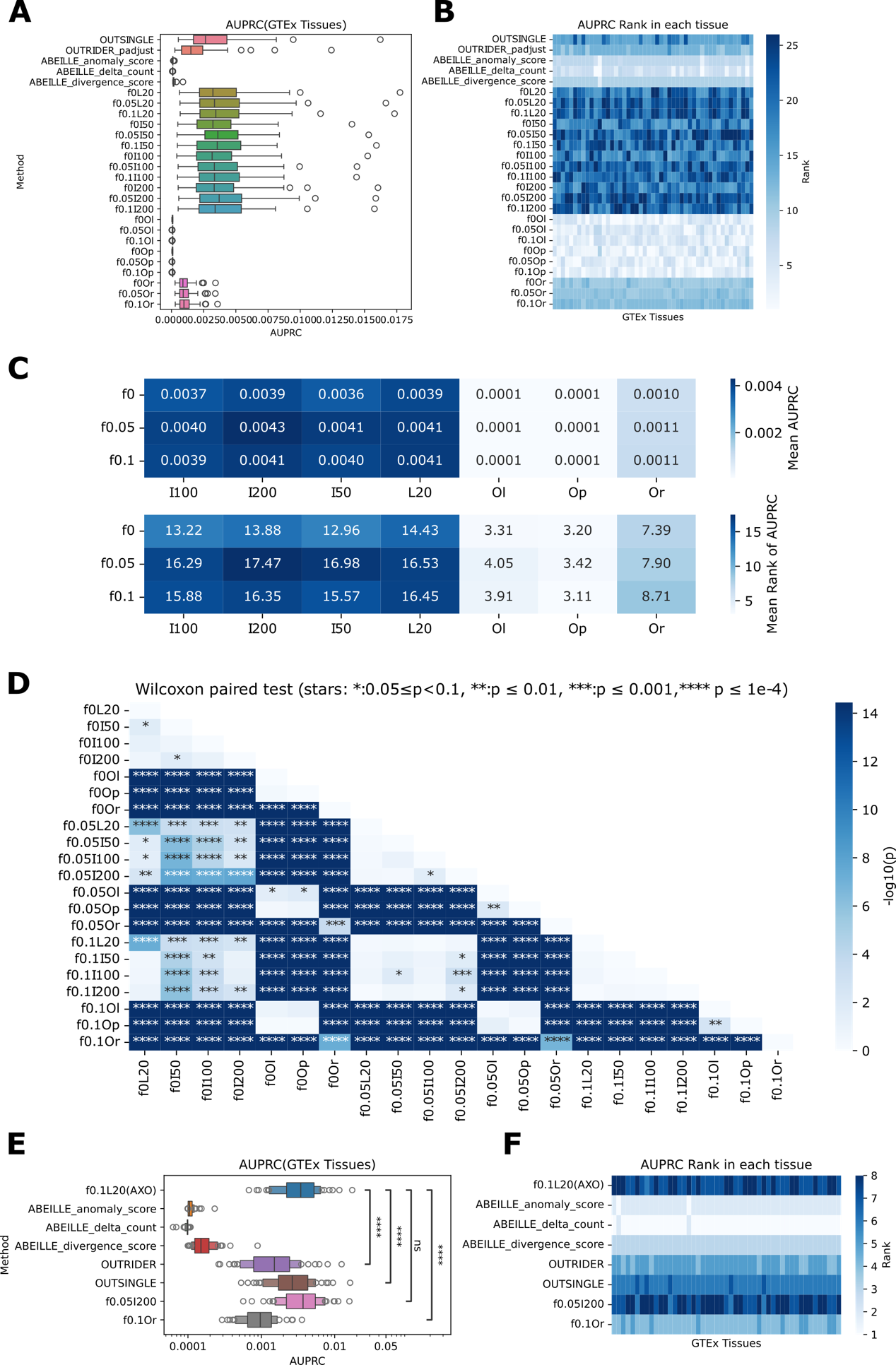
**

**Figure S2.** Performance comparison on the GTEx dataset (49 tissues) following replacement of the Local Outlier Factor (LOF) model in AXOLOTL with Isolation Forest or One-Class SVM.

Modified versions of AXOLOTL were constructed using Isolation Forest with varying n_estimators (I50–I200 denote 50 to 200 estimators) and One-Class SVM with different kernel functions (Or, Op, Ol represent the rbf, poly, and linear kernels, respectively). Additionally, distinct thresholds (f0–f0.1 denote the top 0%–0.1% of coexpressed genes) were used to calculate the feature ord_p_devi. The identifier f0.1L20 corresponds to the final AXOLOTL method, and baseline methods were included in the comparison. (A) Area under the precision-recall curve (AUPRC); (B) Heatmap of AUPRC relative ranks across tissues, sorted in ascending order; (C) Heatmap of average AUPRC across all parameter combinations; (D) p-values from Wilcoxon tests between different parameter settings; (E) Boxplots and (F) heatmaps comparing AXOLOTL, the best-performing Isolation Forest/One-Class SVM models, and baseline methods.


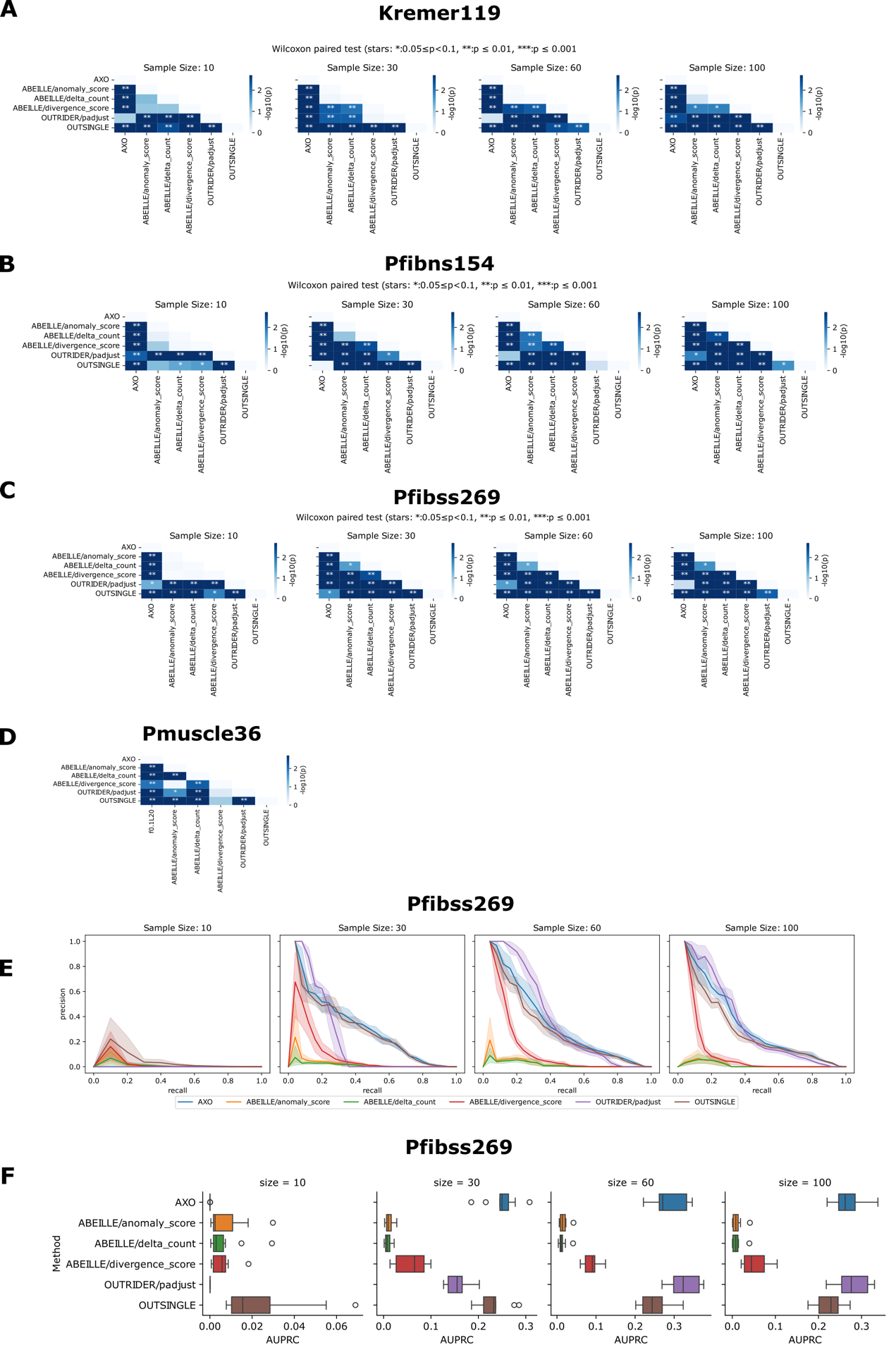


**Figure S3.** Performance comparison of AXOLOTL and baseline methods across multiple datasets and sample sizes.

*p*-values from Wilcoxon paired tests between methods at various cohort sizes on different datasets: (A) Kremer119, (B)Pfibns154, (C) Pfibss269, and (D) Pmuscle36. Detailed performance metrics for the Pfibss269 dataset include: (E) PR curve with 95% CI; (F) boxplot of Area Under the PR curve (AUPRC).

**
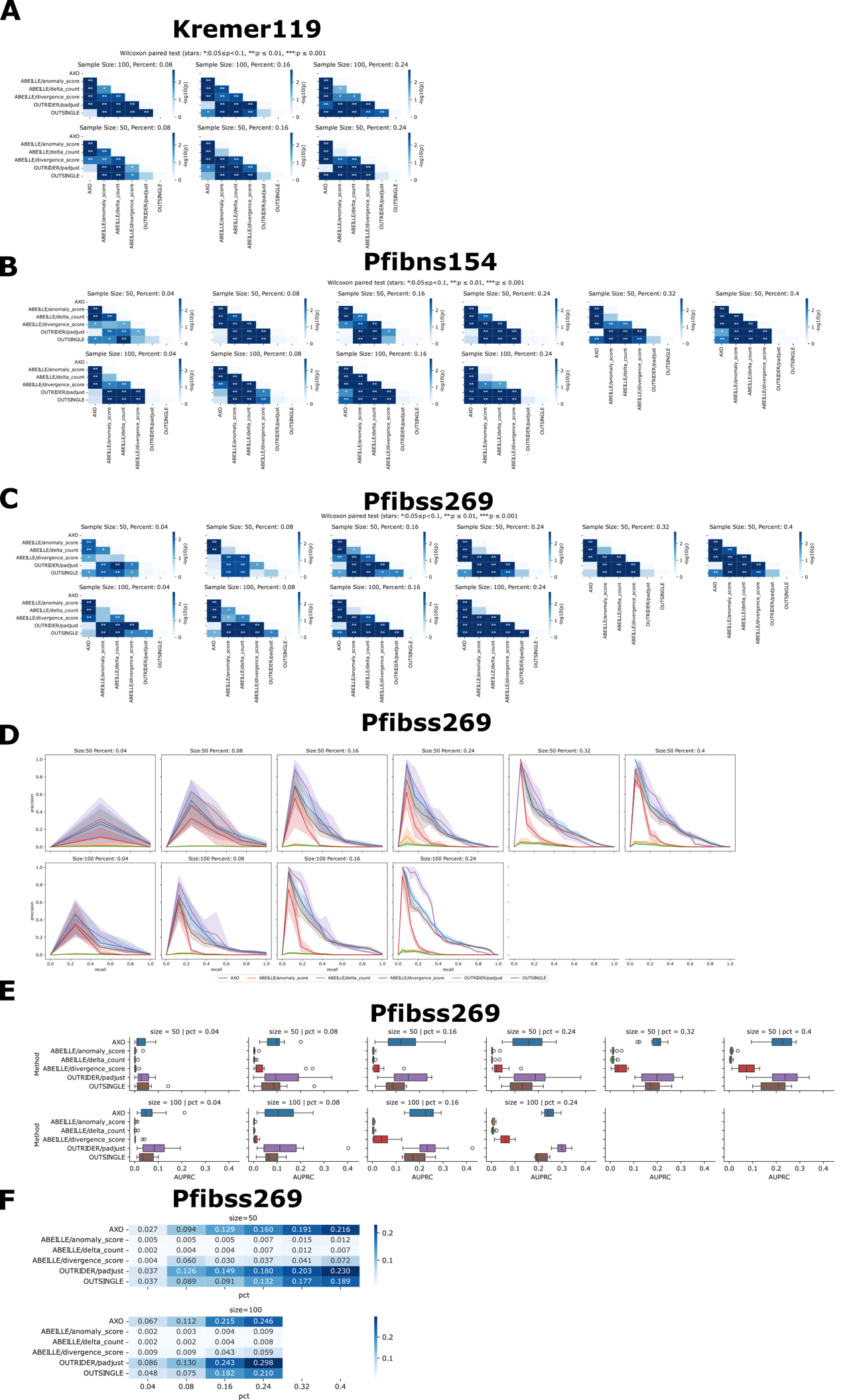
**

**Figure S4.** Robustness analysis of AXOLOTL on public datasets.

*p*-values from Wilcoxon paired tests between methods at various cohort sizes on different datasets: (A) Kremer119, (B)Pfibns154, and (C) Pfibss269. Detailed performance metrics for the Pfibss269 dataset include: (D) PR curve with 95% CI; (E) boxplot of Area Under the PR curve (AUPRC); (F) heatmap of average AUPRC values. Simulations are conducted across sample sizes ranging from 50 to 100 and outlier sample percentages from 4% to 40%.


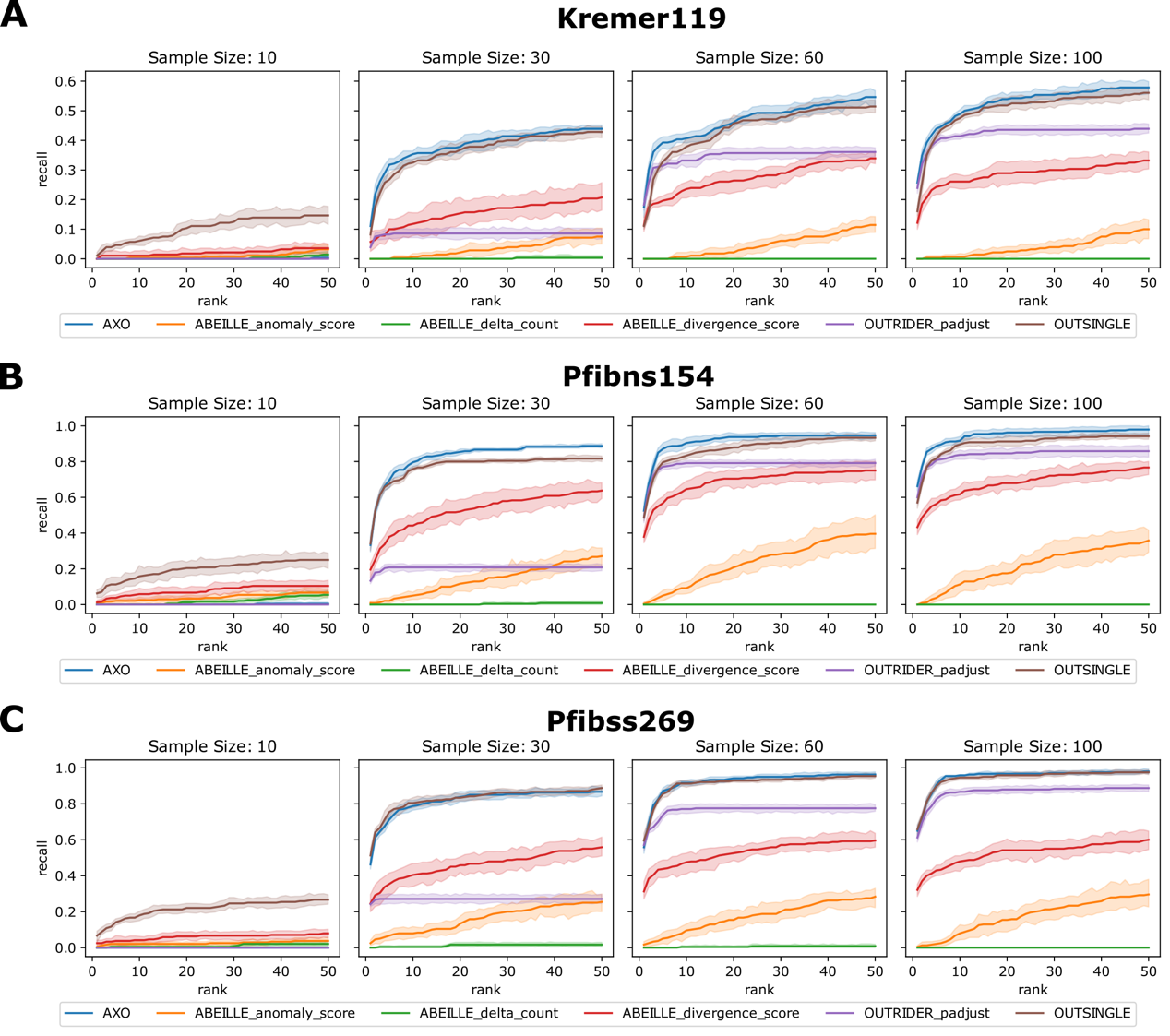


**Figure S5.** Performance comparison of AXOLOTL and baseline methods across multiple datasets and sample sizes.

Top-1~50 hits recall curve with 95% CI: (A) Kremer119, (B)Pfibns154, (C) Pfibss269. Simulations are conducted across sample sizes ranging from 10 to 100.


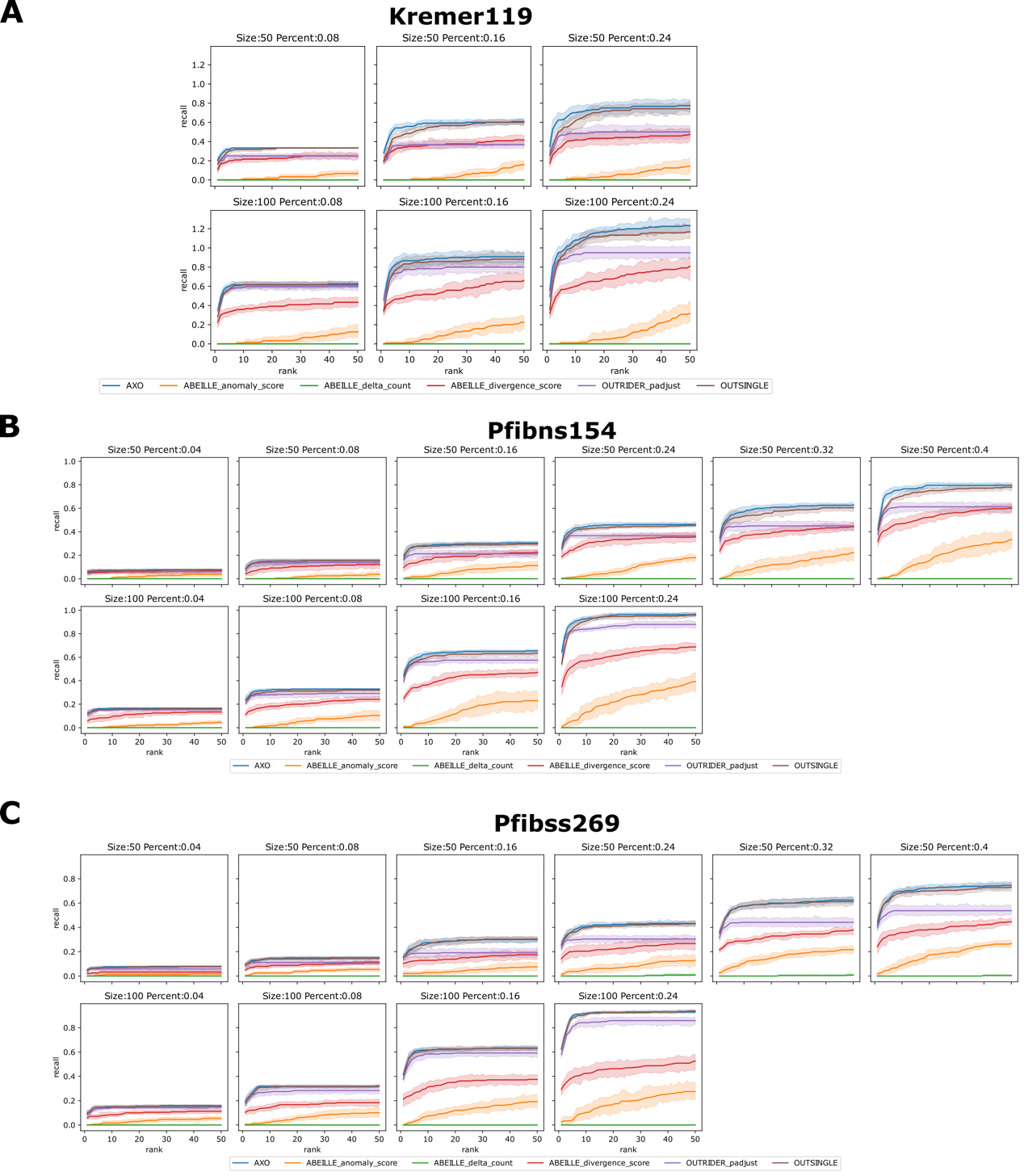


**Figure S6.** Robustness analysis of AXOLOTL on public datasets.

Top-1~50 hits recall curve with 95% CI: (A) Kremer119, (B)Pfibns154, (C) Pfibss269. Simulations are conducted across sample sizes ranging from 50 to 100 and outlier sample percentages from 4% to 40%.


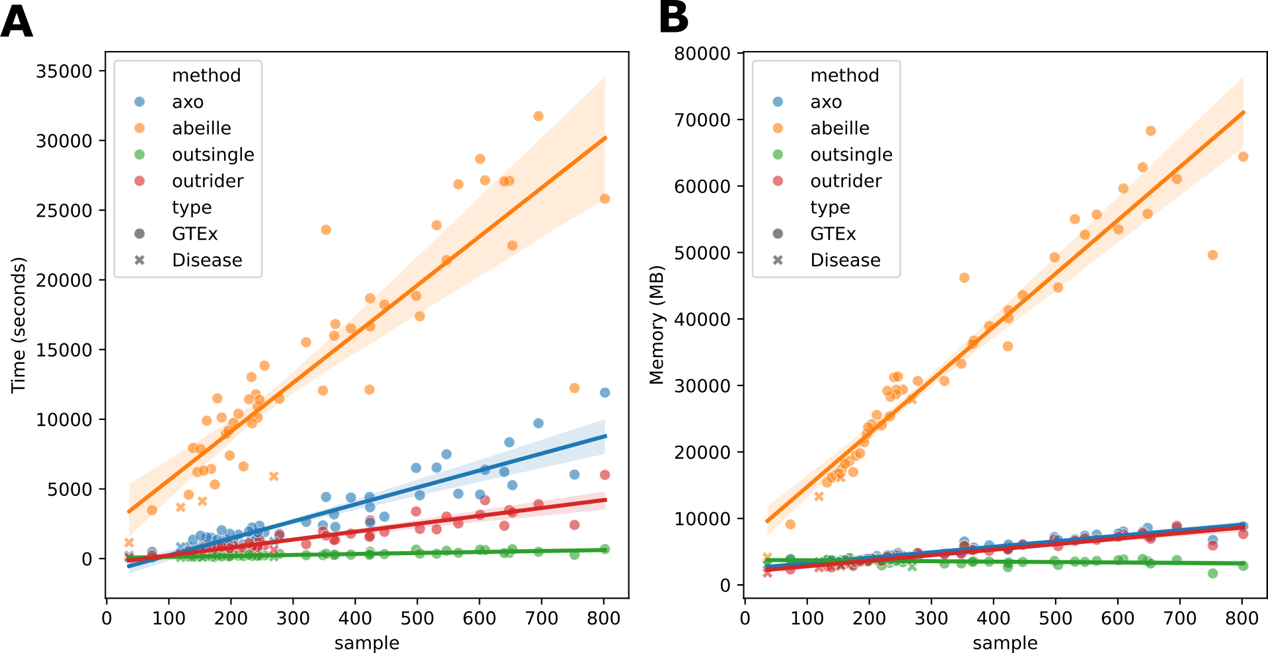


**Figure S7.** Runtime and peak memory usage analysis for AXOLOTL and baseline methods across multiple datasets and sample sizes.

(A) the runtime. The x-axis represents the number of samples and the y-axis denotes computation time in seconds. (B) peak memory. the x-axis represents the number of samples and the y-axis denotes peak memory usage in megabytes. The dot color indicates the method: AXOLOTL (blue), ABEILLE (orange), OutSingle (green) and OUTRIDER (red). The dot shape indicates the dataset type: GTEx (circle) and disease cohorts (cross marks).
